# Hepatitis Awareness Month and Testing Day — May 2019

**DOI:** 10.15585/mmwr.mm6818a1

**Published:** 2019-05-10

May is designated as Hepatitis Awareness Month, and May 19 is Hepatitis Testing Day. Hepatitis B and hepatitis C, the most common types of viral hepatitis in the United States, can cause chronic infections, and many persons remain unaware of their infection until serious complications occur. In 2016, an estimated 862,000 and 2.4 million persons were living with hepatitis B and hepatitis C, respectively, despite availability of a vaccine and effective treatment for hepatitis B and a cure for hepatitis C (*1*,*2*).

Although hepatitis A is preventable through vaccination, multiple states have had outbreaks since 2016, with unprecedented large numbers of cases and person-to-person spread (primarily among persons who use drugs or experience homelessness). A report in this issue of *MMWR* summarizes this resurgence of hepatitis A among unvaccinated adults at risk (*3*).

New cases of hepatitis C are also increasing; during 2010–2016, they increased 3.5-fold, mostly among young adults (*4*). Recent increases in viral hepatitis infections, many attributed to surges in injection-drug use (*4*), highlight the importance of acknowledging and combatting the infectious disease consequences of the nation’s opioid crisis.
